# Supplementary material for: A Method for Estimating 24 h Urinary Sodium and Potassium Excretion by Spot Urine Specimen in Stroke Patients
Source: Nutrients. 2022 Oct 2;14(19):4105. doi: 10.3390/nu14194105 (PMC9573759; doi:10.3390/nu14194105)
Supplement: Supplementary file 1 [file nutrients-14-04105-s001.zip › nutrients-1907759-supplementary.pdf]

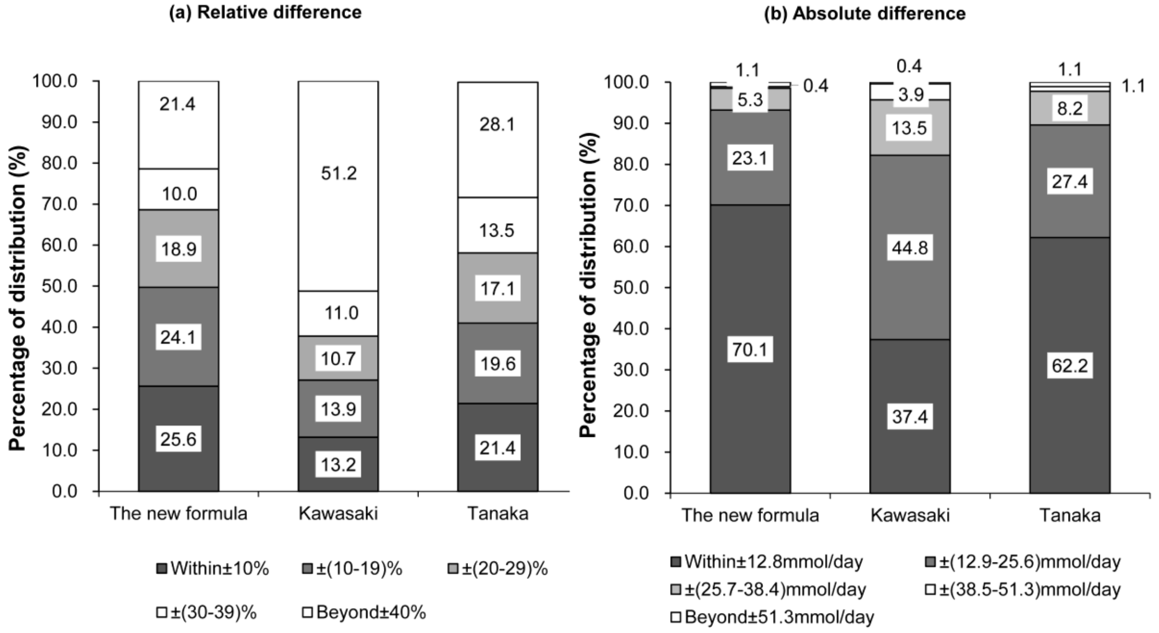

Figure S1: Relative difference and absolute difference distributions of measured and estimated 24-h urinary sodium excretion (24UNaV). Relative difference (a); Absolute difference (b).

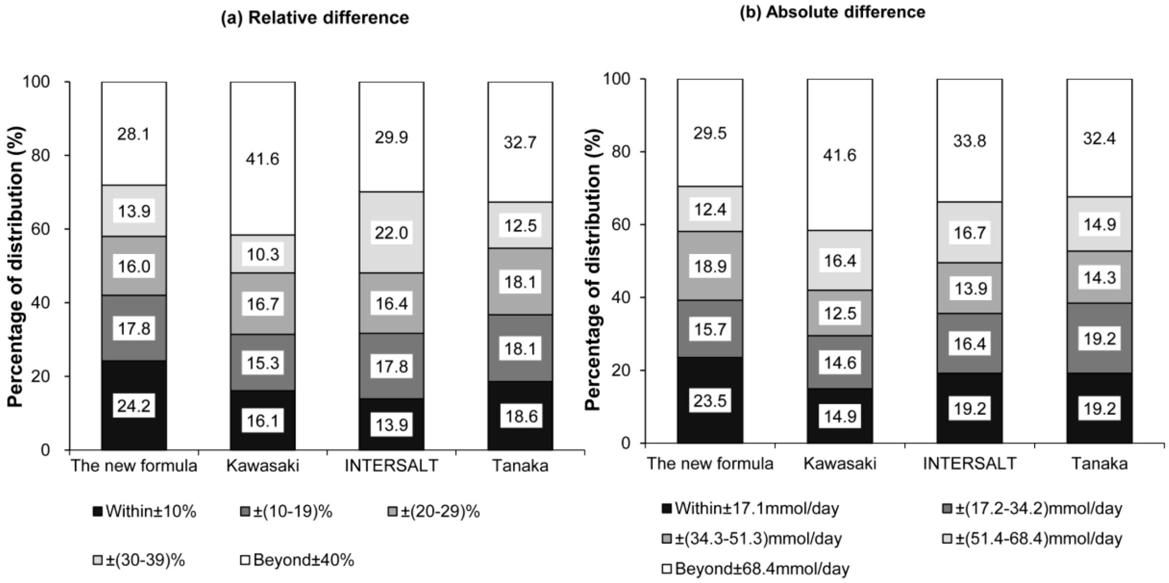

Figure S2: Relative difference and absolute difference distributions of measured and estimated 24-h urinary potassium excretion (24UKV). Relative difference (a); Absolute difference (b).

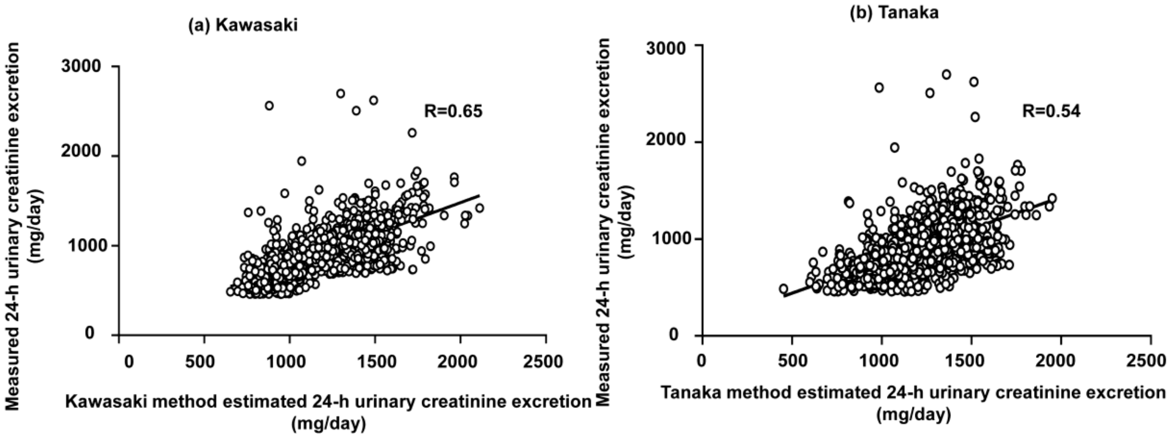

Figure S3: Scatter plots of measured vs. estimated 24 h urinary creatinine excretion (mg/day) by Kawasaki formula (a) and Tanaka formula (b). The hollow circles are scatter points of measured and estimated values. The solid black line is the regression line of the scatters.
